# Circulating CD45^+^EpCAM^+^ cells as a diagnostic marker for early-stage primary lung cancer

**DOI:** 10.3389/fmedt.2022.982308

**Published:** 2022-09-06

**Authors:** Zhen Sun, Peng Li, Zhaojun Wu, Bin Li, Wenjing Li, Mingming Zhao, Xiaobin Zhou, Zeyao Wang, Zhongjie Yu, Wenna Liu, Wenshu Zhu, Haibo Wang, Yongjie Wang

**Affiliations:** ^1^Qingdao Sino-Cell Biomed Co., Ltd., Qingdao, China; ^2^Department of Thoracic Surgery, The Affiliated Hospital of Qingdao University, Qingdao, China; ^3^Department of Epidemiology and Health Statistics, School of Public Health, Qingdao University, Qingdao, China; ^4^Institute of Translational Research for Solid Tumor, Qingdao University, Qingdao, China

**Keywords:** lung cancer, PBMCs, CD45^+^ EpCAM^+^ cells, CEA, diagnosis

## Abstract

Lung cancer is a highly prevalent type of cancer, accounting for 11.6% of all cancer incidences. Early detection and treatment can significantly improve the survival rate and quality of life of patients; however, there is no accurate, effective, and easy-to-use test for early lung cancer screening. In this study, flow cytometry was used to detect the presence of CD45^+^EpCAM^+^ cells in tumor tissues and peripheral blood mononuclear cells (PBMCs) in patients with lung cancer. Moreover, the proportion of CD45^+^EpCAM^+^ cells in PBMCs of patients with lung cancer was found to be significantly higher than that of healthy volunteers. Tumor-related serum markers level was also measured in the peripheral blood of these patients using an electrochemiluminescence assay. The correlation between CD45^+^EpCAM^+^ cells, carcinoembryonic antigen (CEA), and lung cancer was investigated using receiver operating characteristic (ROC) curve analysis, which showed the sensitivity and specificity of the CD45^+^EpCAM^+^ cell to be 81.58% and 88.89%, respectively. Further analysis yielded an area under the ROC curve (ROC/area under the curve [AUC]) of 0.845 in patients PBMCs with lung cancer, which was slightly higher than that of CEA (0.732). Therefore, the detection of CD45^+^EpCAM^+^ cells in PBMCs may be helpful for the early screening and auxiliary diagnosis of lung cancer.

## Introduction

Cancer continues to be a major global public health problem due to its increasing incidence and mortality (1). For a long time, lung cancer has been the leading cause of cancer-related deaths, with a high incidence rate (1, 2). Lung cancer has an insidious development, poor diagnosis, easy metastasis and recurrence, and extremely poor prognosis. Furthermore, 70–75% of patients are detected only at advanced stages, greatly reducing their survival rate. Despite the recent advances in the diagnosis, treatment, and care of patients with lung cancer, most of them have a poor prognosis, with an overall 5-year survival rate of 18.1% because they are at an advanced stage when first diagnosed (3). Therefore, early lung cancer detection will have significant clinical value.

Currently, low-dose computed tomography (LDCT) is considered as a promising method for lung cancer screening (4, 5). The National Lung Screening Trial, first conducted in the United States in 2011, found that LDCT improved the detection rate of patients with early-stage lung cancer, thereby significantly reducing their mortality rate (6). However, early-stage tumor lesions are usually small in size, limiting the sensitivity of imaging techniques. As a result, many lung nodules are evaluated further using radiographic or invasive procedures, resulting in unnecessary radiation exposure, tissue damage, and financial cost. Histopathology is currently the gold standard for tumor diagnosis, but it is not suitable for early screening due to its invasiveness. In addition, serum level of tumor markers, such as carcinoembryonic antigen (CEA), cytokeratin-19 fragment (CYFRA21-1), and neuron-specific enolase (NSE), is commonly used to facilitate diagnosis of lung cancer, but the results are often nonspecific or unreliable; false-positive results are frequently caused by infection, benign tumors, pregnancy, or other factors (7).

Blood-based tests or “liquid biopsies” for circulating tumor cells (CTCs), circulating tumor DNA (ctDNA), circulating microRNAs (miRNAs), and exosomes can be used for the early screening of lung cancer. CTCs are tumor cells that escape from the primary tumor and spread into the bloodstream or lymphatic system (8). Previous studies have shown that CTCs could also cause tumor recurrence and are associated with a poor prognosis (9, 10). Recently, the detection of CTCs has been developed as a diagnostic and prognostic method for patients with lung cancer (11). The migration of CTCs is an early event in cancer progression. In 2009, Tanaka et al. reported the first detailed study on the diagnostic significance of CTCs in patients with suspected or diagnosed primary lung cancer using the CellSearch system (12). Despite many CTCs isolation and enrichment techniques available for clinical studies, the small number of CTCs in the circulating blood results in drawbacks, such as large sampling volume, poor sensitivity, low specificity, cumbersome operation, and high cost. ctDNA has become a compelling method that allows clinicians to repeatedly study the dynamic evolution of malignant tumors in a non-invasive manner (13, 14). As tumor cells die, ctDNA is released into the bloodstream in small amounts, which represents a promising strategy for cancer surveillance (15). The detection of ctDNA is also associated with the risk of recurrence and long-term prognosis of patients with lung cancer after surgery (15, 16). Other blood circulation markers, such as proteins, antibodies, miRNAs, and exosomes, are also under investigation (17–19). However, there is still no non-invasive, simple, and rapid test for the early screening or auxiliary diagnosis of lung cancer.

Ishizawa et al. reported the presence of CD45^+^EpCAM^+^ cells in both solid tumor tissues and malignant pleural effusions of patients with non-small cell lung cancer (NSCLC); it is highly suspected that cell population is involved in epithelial–mesenchymal transition (20). CD45^+^EpCAM^+^ cells are also insensitive to widely used drug regimens, more invasive, and able to avoid natural killer cell-mediated immune surveillance in human epithelial ovarian cancer (EOC) (21). Recently, we reported that the formation of CD45^+^EpCAM^+^ cells in patients with lung cancer is associated with tumor cell-derived exosomes (22). To the best of our knowledge, the presence of CD45^+^EpCAM^+^ cells in PBMCs of patients with lung cancer has not been reported yet. Our current study reported the presence of CD45^+^EpCAM^+^ cells not only can be detected in the tumor tissue of patients but also in the PBMCs of these patients. Moreover, CD45^+^EpCAM^+^ cells in patients' PBMCs were significantly higher than that in healthy volunteers. Further analysis of various serum tumor markers demonstrated that the detection of CD45^+^EpCAM^+^ cells in the circulating blood has a higher specificity for the diagnosis of lung cancer than commonly used serological auxiliary tests. This method is easy to perform, economical, rapid, and can be easily promoted in a large area; it is expected to be an auxiliary method for the early screening and diagnosis of lung cancer.

## Materials and methods

### Human subjects

In this study, all patients with lung cancer were treated at the Affiliated Hospital of Qingdao University between 2021 and 2022. This study was approved by the Medical Ethics Committee of Affiliated Hospital of Qingdao University under the registration number QYFYKYLL920511921. The histopathological diagnosis of these patients was performed by at least two pathologists in accordance with the classification of the World Health Organization. There was no history of cancer or antitumor treatment prior to the initial diagnosis. All patients with lung cancer were pathologically or cytologically confirmed, including 35 cases of adenocarcinoma, and 1 case of SCLC, and 1 case of squamous cell carcinoma, and 1 case unidentified. The control group consisted of 42 healthy volunteers, 25 females and 17 males, ages from 25 to 48.

### Acquisition of single-cell suspensions of lung cancer tumor tissues

As described below, tumor tissues were obtained from patients with lung cancer using minimally invasive surgery, and a single-cell preparation was immediately carried out. Firstly, fresh lung tumor samples were cut into 1–3-mm^3^ pieces, added to an appropriate amount of RPMI-1640 medium containing 10% fetal bovine serum (FBS), and gently grounded on a 40-mm cell filter with the plunger of a 20-ml syringe until a homogeneous cell suspension was obtained. Then, the cell suspension was filtered using a cell filter and centrifuged at 400 g for 10 min. After washing twice with 1× phosphate-buffered saline (PBS), the cell precipitate was re-suspended in RPMI-1640 medium containing 10% FBS.

### Extraction of PBMCs

PBMCs were isolated from the peripheral blood (5–10 ml) of all subjects using density reagents. After centrifugation at a 700 g density gradient for 30 min, PBMCs settled in interphase precipitation were carefully collected and washed twice with 1× PBS. Cell precipitates were resuspended in RPMI-1640 medium containing 10% human AB serum, and 1% penicillin and 1% streptomycin.

### Flow cytometry assay

In this study, single-cell suspensions for surface staining were prepared from the subjects' tumor tissues or peripheral blood. Anti-human EpCAM (9C4), anti-human CD45 (2D1), anti-human CD3 (OKT3), anti-human CD19 (4G7), and anti-human CD16 (3G8) (BioLegend) were used as fluorochrome-coupled antibodies. Flow cytometry data were collected using CytoFLEX (Beckman-Coulter, Fullerton, CA, USA) and analyzed with FlowJo software (TreeStar).

### Detection of serum biomarkers

Serum was collected from the patients and assayed for cancer biomarkers, including ProGRP, SCC, CEA, NSE, CYFRA21-1 and CA125, using electrochemiluminescence, with normal upper detection limits of 63 pg/ml, 2.5 ng/ml, 3.4 ng/ml, 17 ng/ml, 3.3 ng/ml, and 35 U/ml, respectively. All serum samples were collected and detected by the clinical laboratory of Qingdao University Hospital.

### ROC/AUC analysis

Statistical analysis was performed using MedCalc statistical software to generate patients' ROC curves and calculate the area under the curve (AUC) to compare the diagnostic accuracy of the detected markers in predicting lung cancer.

### Statistical analysis

Using the GraphPad Prism software (Version 8.0), the statistical significance of differences between the groups was determined using one-way analysis of variance (ANOVA) followed by Tukey test, *P* < 0.05 were considered statistically significant.

## Results

### Patient and tumor characteristics

The clinicopathologic characteristics of 38 patients with lung cancer are shown in Table 1. Their ages ranged from 45 to 78, and 47.4% were men. Thirty-five patients had adenocarcinomas (92.2%), one had small cell lung cancer (SCLC) (2.6%), one had squamous cell carcinoma (2.6%), one had lung tumor (2.6%). Of the patients with lung cancer, 1 (2.6%) had tumor stage 0, 15 (39.5%) had tumor stage I, 6 (15.8%) had tumor stage II, and 5 (13.2%) had tumor stage III. According to the Eastern Cooperative Oncology Group (EGOC) score, 33 patients (86.8%) had an EGOC score of 0, while 5 patients (13.2%) had a score of 1.

**Table 1:** Clinicopathological characteristics of patients with lung cancer.

	**No. of patients (n** ** = 38)**	**%**
**Age**	45-78	
**Gender**		
Male Female	18 20	47.4 52.6
**Histopathology**		
Adenocarcinoma SCLC Squamous cell carcinoma Unidentified	35 1 1 1	92.2 2.6 2.6 2.6
**Stage**		
0 I II III No Information	1 15 6 5 11	2.6 39.5 15.8 13.2 28.9
**ECOG PS**		
0 1	33 5	86.8 13.2
**Smoking**		
Yes No	10 28	26.3 73.7

PS, performance status.

### CD45^+^EpCAM^+^ cells were detected in PBMCs of patients with lung cancer

**Figure 1:**
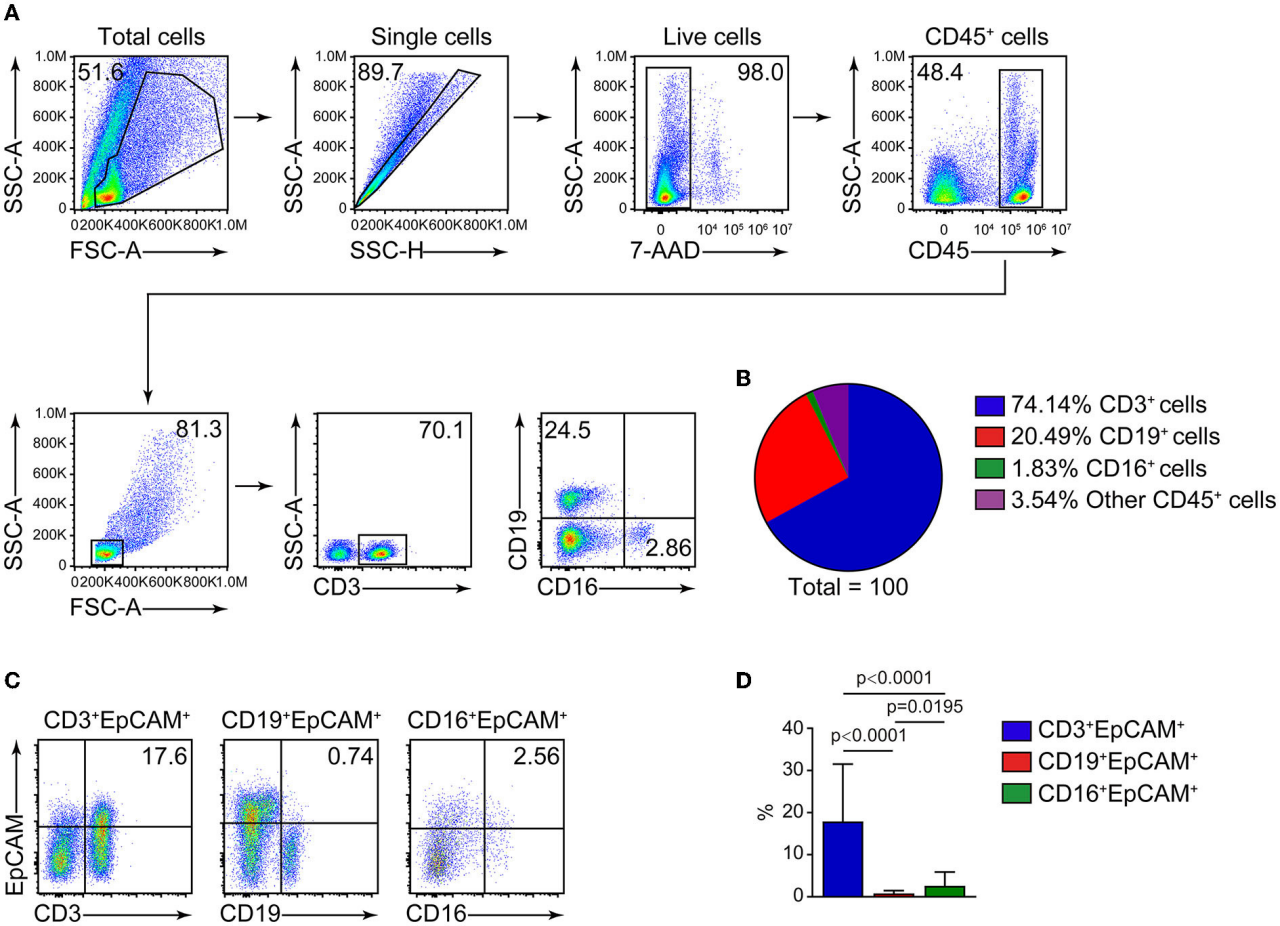
FACS analysis of cells in lung cancer tumor tissues. **(A)** Representative flow cytometry plots of CD3^+^, CD19^+^, CD16^+^ cells analysis from tumor cells of patients with lung cancer. **(B)** Summary of the ratio of CD3^+^, CD19^+^, CD16^+^ cells (*n* = 29). **(C)** Flow cytometry analysis of CD3^+^EpCAM^+^, CD19^+^EpCAM^+^, CD16^+^EpCAM^+^ cell ratio (gated on 7AAD^−^CD45^+^ cells) in tumor cells from patients with lung cancer. **(D)** Quantification data on the ratio of CD3^+^EpCAM^+^, CD19^+^EpCAM^+^, CD16^+^EpCAM^+^ cells (*n* = 29).

**Figure 2:**
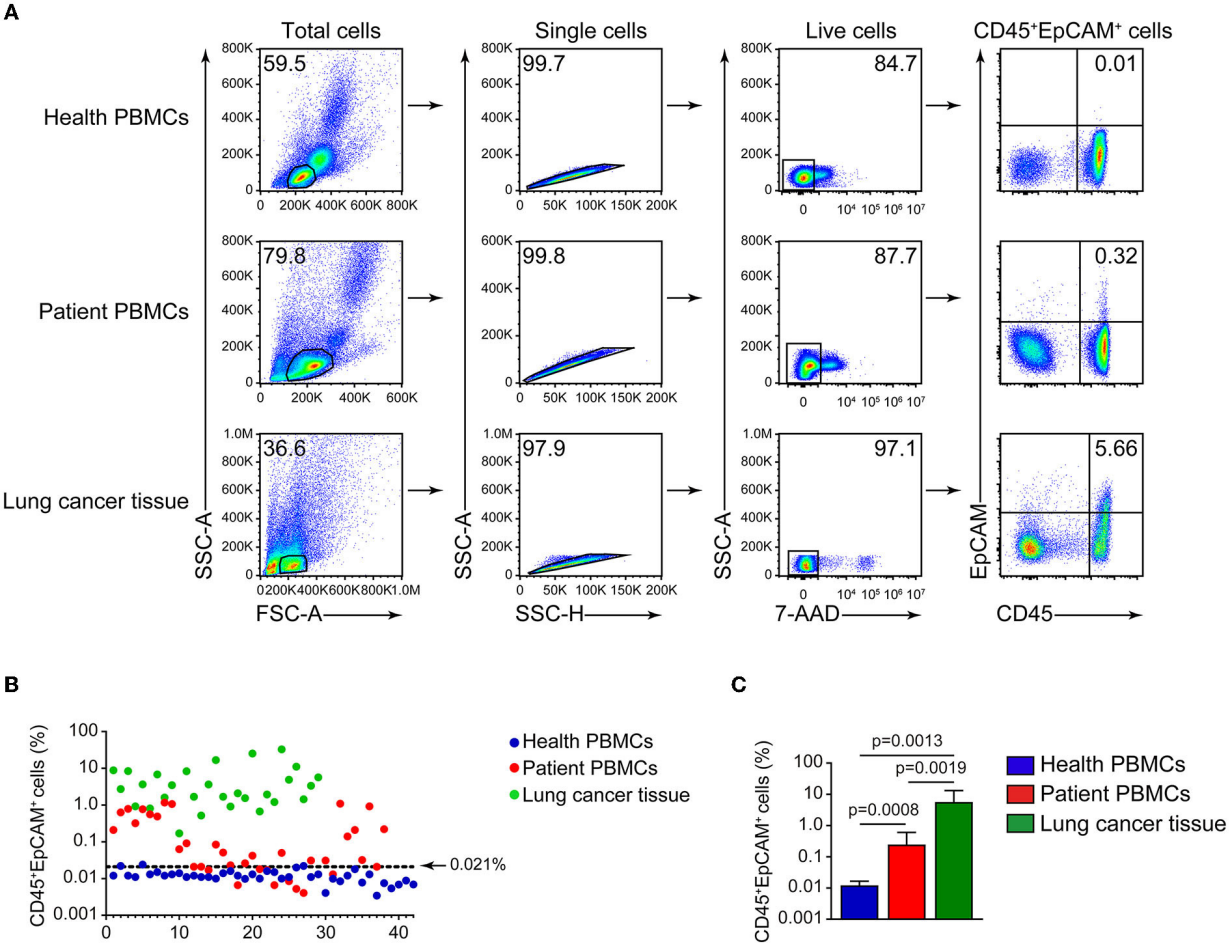
Detection of CD45^+^EpCAM^+^ cells in lung cancer tumor tissues and PBMCs. **(A)** Representative flow cytometry plots of CD45^+^EpCAM^+^ cells proportion in PBMCs of healthy volunteers, patients' PBMCs and lung cancer. **(B)** Depiction of CD45^+^EpCAM^+^ cell ratio difference in PBMCs of healthy volunteers, patients' PBMCs and lung tumors (healthy volunteers = 42, patients = 38, lung tumors = 29). The top limit for a normal range (0.021) was determined by healthy volunteer's mean + 2 SD. **(C)** Statistical plots of CD45^+^EpCAM^+^ cell ratio in PBMCs of healthy volunteers, patients' PBMCs and lung cancer cells (healthy volunteers = 42, patients = 38, lung tumors = 29).

CD45 is a leukocyte marker, whereas EpCAM is an epithelial cell marker. In lung cancer, CD45^+^ cells are mostly harboring lymphocyte markers CD3 and CD19 (Figures 1A,B), and the proportion of CD3^+^EpCAM^+^ cells is higher than that of CD19^+^EpCAM^+^ and CD16^+^EpCAM^+^ cells (Figures 1C,D). We found the presence of CD45^+^EpCAM^+^ cells in the tumor tissues of all 38 patients with lung cancer by flow cytometry. Then, we asked the question if CD45^+^EpCAM^+^ cells also presents in the peripheral blood of these lung cancer patients. Since most of the immune cells in tumor microenvironments are lymphocytes, we used FSC and SSC to gate out the lymphocytes for further analysis. Our results showed CD45^+^EpCAM^+^ cells were also present in lung cancer patients' PBMCs (Figure 2A). Then, we want to study if the detection of CD45^+^EpCAM^+^ cells in PBMCs is specific for lung cancer patients. PBMCs of 42 healthy volunteers were examined, revealing that the ratio of CD45^+^EpCAM^+^ in PBMCs of patients with lung cancer was significantly higher than that of healthy volunteers (patients 0.244 ± 0.353 vs. heath volunteers 0.012 ± 0.004, presented as mean ± *SD*, Figures 2B,C).

Then, we measured the commonly used cancer auxiliary test of six serum cancer markers in patients. As shown in Table 2, there are respectively 2 (5.3%), 1 (2.6%), 11 (28.9%), 2 (5.3%), and 4 (11.1%) patients showed serum level above the normal range for ProGRP, SCC, CEA, NSE and CYFRA21-1. All patients' serum CA125 levels were in normal range. CEA is a human embryonic antigen-specific determinant acid glycoprotein, which is a non-organ-specific tumor-associated antigen, and that is found in very low level in normal adults' circulating blood. Elevated level of CEA correlates closely with tumor cell proliferation and is widely used in various tumor tests (23, 24). Then we analyzed CD45^+^EpCAM^+^ ratio in PBMCs of patients and health volunteers. Using 42 health volunteers' mean plus two standard deviations as the top limit (0.021), we found there are 28 patients (73.7%) were above this top limit while only 3 health volunteers (7.1%) were above this top limit. However, only 11 patients (28.9%) showed increased CEA levels (Table 3).

**Table 2:** Six cancer markers serum level in patients with lung cancer.

	**No. of patients** **(n ≤ 38)**	**%**
**ProGRP (pg/ml)**		
Median (range) Normal (< 63) Elevated (≥ 2.5)	31.45 (22.67-96.06) 36 2	94.7 5.3
**SCC (ng/ml)**		
Median (range) Normal (<2.5) Elevated (≥ 2.5)	1.01 (0.44-3.79) 37 1	97.4 2.6
**CEA (ng/ml)**		
Median (range) Normal (< 3.4) Elevated (≥ 3.4)	2.12 (0.36-16.10) 27 11	71.1 28.9
**NSE (ng/ml)**		
Median (range) Normal (< 17) Elevated (≥ 17)	12.00 (7.69-19.40) 36 2	94.7 5.3
**CYFRA 21-1 (ng/ml)**		
Median (range) Normal (< 3.3) Elevated (≥ 3.3)	1.78 (0.69-9.18) 32 4	88.9 11.1
**CA125 (U/ml)**		
Median (range) Normal (< 35) Elevated (≥ 35)	9.32 (4.06-28.4) 36 0	100 0

ProGRP, serum progastrin-releasing peptide precursor; SCC, squamous cell carcinoma-associated antigen; CEA, carcinoembryonic antigen; NSE, neuron-specific enolase; CYFRA21-1, cytokeratin-19 fragment; CA125, carbohydrate antigen 125.

**Table 3:** Comparative analysis of CEA serum level and CD45^+^EpCAM^+^ cell ratio in PBMCs from health volunteers and lung cancer patients.

	**No. of change**	**%**
**CD45**^**+**^**EpCAM**^**+**^ **cell (%)**		
Healthy volunteers (n = 42)		
< 0.021 ≥ 0.021	39 3	92.9 7.1
Lung cancer patients (n =38)		
< 0.021 ≥ 0.021	10 28	26.3 73.7
**CEA (ng/ml)**		
Healthy volunteers (n = 42)		
< 3.4 ≥ 3.4	40 2	95.2 4.8
Lung cancer patients (n = 38)		
> 3.4 ≥ 3.4	27 11	71.1 28.9

### Diagnostic accuracy of CD45^+^EpCAM^+^ cell ratio in PBMCs for lung cancer

**Figure 3:**
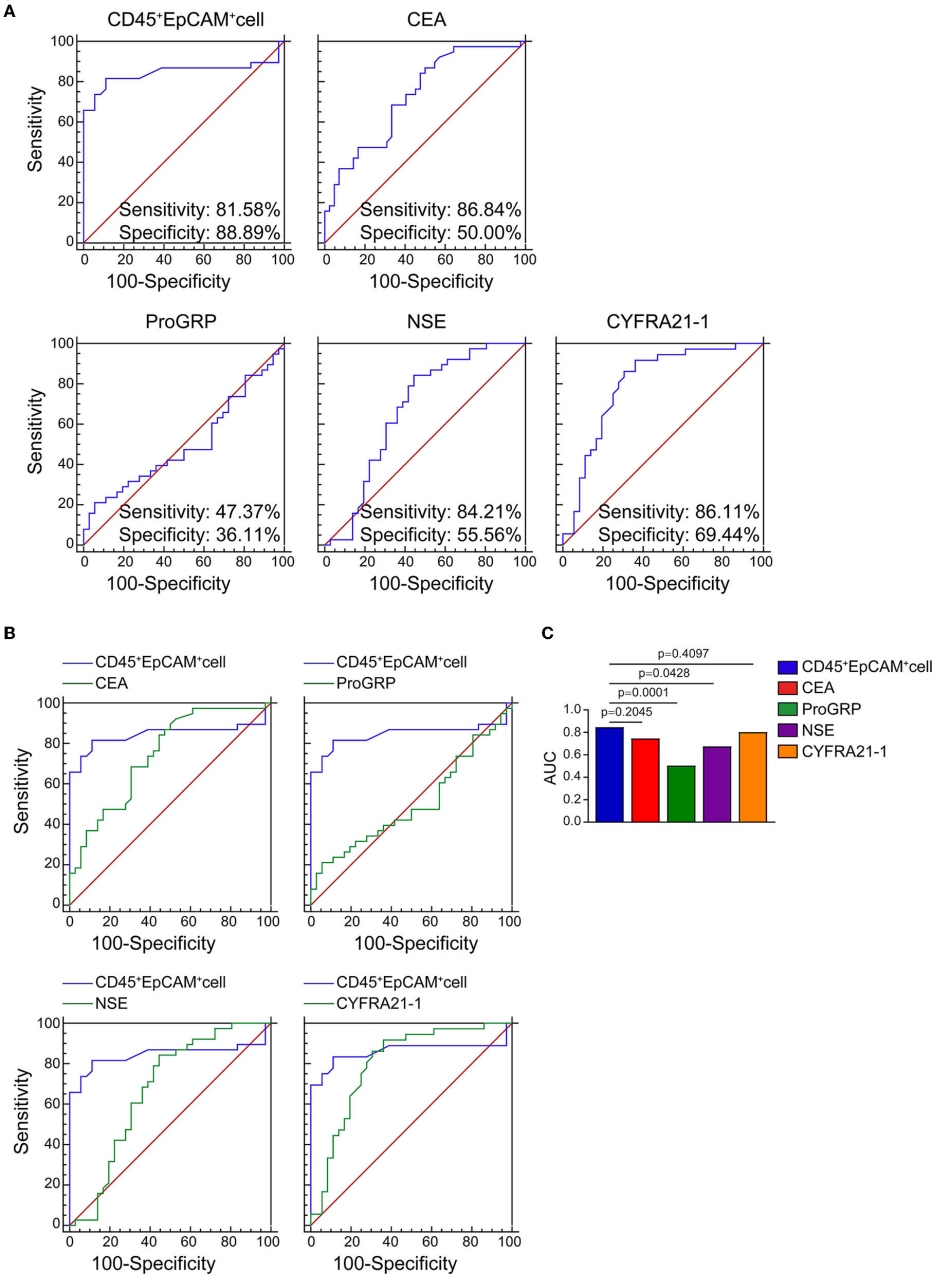
ROC and AUC analysis of CEA, ProGRP, NSE, CYFRA21-1 serum level serum level and CD45^+^EpCAM^+^ cell ratio in PBMCs of lung cancer patients. **(A)** ROC curve analysis of CEA, ProGRP, NSE, CYFRA21-1 serum level and CD45^+^EpCAM^+^ cell ratio (healthy volunteers = 42, patients = 38). (healthy volunteers = 42, patients = 38). **(B)** The comparison of CEA, ProGRP, NSE, CYFRA21-1 serum level with CD45^+^EpCAM^+^ cell ratio in AUC analysis. (healthy volunteers = 36, patients = 38). **(C)** Statistic summary of AUC analysis of CEA, ProGRP, NSE, CYFRA21-1 serum level and CD45^+^EpCAM^+^ cell ratio in PBMCs from health volunteers and lung cancer patients (healthy volunteers = 36, patients = 38).

As shown in Table 2, there are 2 (5.3%), 1 (2.6%), 11 (28.9%), 2 (5.3%), 4 (11.1%), and 0 patients showed serum level above the normal range for ProGRP, SCC, CEA, NSE, CYFRA21-1, and CA125. Therefore, using the area under the ROC curve (ROC/AUC) analysis, the sensitivity, specificity, and accuracy of CEA, ProGRP, NSE, CYFRA21-1, and the ratio of CD45^+^EpCAM^+^ cells in PBMCs for the diagnosis of lung cancer were analyzed. The ROC curve showed that the sensitivity/specificity of CEA, ProGRP, NSE and CYFRA21-1 for the diagnosis of lung cancer were 86.84%/50.00%, 47.37%/36.11%, 84.21%/55.56%, and 86.11%/69.44%, respectively (Figure 3A). The sensitivity and specificity of CD45^+^EpCAM^+^ cell ratio in PBMCs of lung cancer patients were 81.58% and 88.89%, respectively (Figure 3A). Further analysis revealed that the AUC of the ratio of CD45^+^EpCAM^+^ cells in PBMCs of patients with lung cancer was 0.845, which was slightly higher to that of serum CEA level (0.732) (Figures 3B,C). The AUC of the ratio of CD45^+^EpCAM^+^ cells in PBMCs of lung cancer patients was significantly higher than that of ProGRP (0.503), NSE (0.674) serum level, and was not different from serum CYFRA21-1 level (0.801) (Figures 3B,C). Information of ROC/AUC analysis of serum biomarker level and CD45^+^EpCAM^+^ cell ratios in PBMCs of lung cancer patients are shown in Table 4. This result further suggested that the changes in the ratio of CD45^+^EpCAM^+^ cells in PBMCs may be useful as an auxiliary method for early lung cancer screening. In clinical practice, the application of the changes in CD45^+^EpCAM^+^ cell ratio of PBMCs combined with the changes in CEA and CYFRA21-1serum level and other diagnostic methods may be useful for an early diagnosis of lung cancer.

**Table 4:** Statistical analysis of ROC/AUC for serum biomarkers' level and CD45^+^EpCAM^+^ cell ratio in PBMCs of healthy volunteers and lung cancer patients.

**Biomarkers**	**Threshold value**	**Sensitivity**	**Specificity**	**AUC (95%CI)**	**P**
CD45^+^EpCAM^+^ cell	0.016	81.58%	88.89%	0.845 (0.743, 0.919)	0.0001
CEA	1.1	86.84%	50.00%	0.732 (0.621, 0.825)	0.0001
ProGRP	32	47.37%	36.11%	0.503 (0.384, 0.621)	0.9661
NSE	9.97	84.21%	55.56%	0.675 (0.555, 0.778)	0.0086
CYFRA21-1	1.25	86.11%	69.44%	0.801 (0.690, 0.886)	0.001

## Discussion

Early diagnosis and treatment are critical for improving the postoperative survival of patients with lung cancer. A simple, economical, and easy-to-promote early screening method will play a key role in its early detection to provide timely treatment. Currently, LDCT cannot identify benign and malignant lung nodules and requires expensive equipment, which is difficult to promote in large area. CTCs and ctDNA are the most prominent components of liquid biopsies and are the focus of current research. Although they have the advantages of being non-invasive, convenient, and reproducible, their low detection rate, sensitivity, and specificity limit their development. Therefore, there is an urgent need to develop a method for early lung cancer screening that is simple to use, has high sensitivity, and is low in cost.

The presence of CD45^+^EpCAM^+^ cells was reported in solid tumor tissues and malignant effusions of patients with NSCLC and EOC, and our previous study showed exosomes from lung cancer cells fused with CD45^+^ cells and the micorRNA from the exosomes induced gene expression change that results in EpCAM expression and also making these CD45^+^EpCAM^+^ cells prone to apoptosis (20–22). However, no studies reported the detection of CD45^+^EpCAM^+^ cells in PBMCs of patients with lung cancer or other cancers. To the best of our knowledge, the present study was the first to report the presence of CD45^+^EpCAM^+^ cells in PBMCs of patients with lung cancer, which was detected using a simple FACS. This was also the first study that explored the possibility of using the changes in CD45^+^EpCAM^+^ cells in PBMCs as a biomarker for early lung cancer screening. CD45^+^EpCAM^+^ cells were detected in the tumor tissues of all 38 patients with lung cancer, and an increased CD45^+^EpCAM^+^ cell ratio was present in PBMCs of 28 patients (73.7%) of these patients. Six others commonly used clinical tumor serologic tests were compared, with CEA being the most sensitive. Abnormal CEA increases in 11 out of 38 patients (28.9%) were observed. ROC curve analysis indicated that the sensitivity and specificity of serum CEA for detecting lung cancer were 86.84% and 50.00%, while the sensitivity and specificity of the CD45^+^EpCAM^+^ cell ratio were 81.58% and 88.89%, respectively. Comparing the two, the specificity of CD45^+^EpCAM^+^ cell ratio for detecting lung cancer was higher. Further analysis using ROC curves showed that the AUC of CD45^+^EpCAM^+^ cell ratio in PBMCs of patients with lung cancer was 0.845, which was slightly higher than that of serum CEA level changes (0.732).

In a study of the correlation between the CTCs detection method and lung cancer tumor staging, the positivity rate of CTCs was higher in patients with stage IV NSCLC than in those with tumor stage III (25). In this study, CD45^+^EpCAM^+^ cell ratio in PBMCs of patients with stage I lung cancer was significantly higher than that of patients with tumor stage II and III. Due to the small sample size (patients' number were 15, 6 and 5 for lung cancer stage I, II, and III respective), this result need to be further validated with increased sample pool. Meanwhile, in this study, we presumed the data as normal distribution and used health volunteer's mean plus 2 standard deviations as the up limit to evaluate if the patient's CD45^+^EpCAM^+^ cell ratio in PBMCs is above “normal range.” We think that future studies with increased both health and patient's sample size are necessary to establish a normal range.

In summary, our results suggest the increase of CD45^+^EpCAM^+^ cell ratio in PBMCs may be useful for early stage lung cancer diagnosis. The advantage of this easy, quick, non-invasive and economic flow-based assay makes this approach valuable for lung cancer screening. Future studies with larger patient population and clinical follow up will contribute more information on the significance of CD45^+^EpCAM^+^ cell elevation in cancer patients' PBMCs.

## Data availability statement

The original contributions presented in the study are included in the article/supplementary materials, further inquiries can be directed to the corresponding author/s.

## Ethics statement

This study was approved by the Medical Ethics Committee of Affiliated Hospital of Qingdao University under the registration number QYFYKYLL920511921. The patients/participants provided their written informed consent to participate in this study.

## Author contributions

ZS performed and analyzed all experiments and were involved in drafting the manuscript. PL provided peripheral blood and tumors from patients with lung cancer. ZWu was responsible for extracting PBMCs. BL, MZ, ZWa, WLi, ZY, WLiu, and WZ assisted with the total experiments. XZ provided support for data analysis. HW and YW supervised the project and designed the experiments. All authors have read and endorsed the ultimate manuscript.

## Funding

This work was supported by grants from the Wu Jieping Medical Foundation (320.6750.2021-01-4 to YW) and the Project for Clinical Medicine + X supported by the Affiliated Hospital of Qingdao University (QYFY-X2021032/3731 to YW).

## Conflict of interest

ZS, ZWu, BL, WLi, MZ, ZWa, ZY, WLiu, and WZ are employed by Sino-Cell Biomed Co., Ltd. The remaining authors declare that the research was conducted in the absence of any commercial or financial relationships that could be construed as a potential conflict of interest.

## Publisher's note

All claims expressed in this article are solely those of the authors and do not necessarily represent those of their affiliated organizations, or those of the publisher, the editors and the reviewers. Any product that may be evaluated in this article, or claim that may be made by its manufacturer, is not guaranteed or endorsed by the publisher.
